# Understanding the combining ability for physiological traits in soybean

**DOI:** 10.1371/journal.pone.0226523

**Published:** 2019-12-17

**Authors:** Larissa Pereira Ribeiro Teodoro, Leonardo Lopes Bhering, Bruno Ermelindo Lopes Gomes, Cid Naudi Silva Campos, Fabio Henrique Rojo Baio, Ricardo Gava, Carlos Antonio da Silva Júnior, Paulo Eduardo Teodoro

**Affiliations:** 1 Department of Plant Science, Universidade Federal de Mato Grosso do Sul, Chapadão do Sul, Mato Grosso do Sul, Brazil; 2 Department of General Biology, Universidade Federal de Viçosa, Viçosa, Minas Gerais, Brazil; 3 Department of Geography, Universidade do Estado do Mato Grosso, Sinop, Mato Grosso, Brazil; University of Guelph, CANADA

## Abstract

Photosynthetic efficiency has become the target of several breeding programs since the positive correlation between photosynthetic rate and yield in soybean suggests that the improvement of photosynthetic efficiency may be a promising target for new yield gains. However, studies on combining ability of soybean genotypes for physiological traits are still scarce in the literature. The objective of this study was to estimate the combining ability of soybean genotypes based on F_2_ generation aiming to identify superior parents and segregating populations for physiological traits. Twenty-eight F_2_ populations resulting from partial diallel crossings between eleven lines were evaluated in two crop seasons for the physiological traits: photosynthesis, stomatal conductance, internal CO_2_ concentration, and transpiration. General combining ability (GCA) of the parents and specific combining ability (SCA) of the F_2_ populations were estimated. Our findings reveal the predominance of additive effects in controlling the traits. The genotype TMG 7062 IPRO is the most promising parent for programs aiming at photosynthetic efficiency. We have also identified other promising parents and proposed cross-breeding with higher potential for obtaining superior lines for photosynthetic efficiency.

## 1. Introduction

Soybean [*Glycine max* (L.) Merril] is the most economically important oilseed in the world, whose yield has grown considerably in the last three decades. Among the factors that contributed to this scenario, we highlight the genetic breeding [1–4]. In breeding programs aiming at obtaining high yielding genotypes, the evaluation of genetic diversity to identify the crosses that provide more significant heterotic effect is an essential step, since it increases the probability of obtaining transgressive segregating progenies [5]. Among the methods based on biometric models for evaluating the diversity of parents, there are diallel crosses.

Diallel crosses provide information about the genetic control of the traits evaluated, which helps in conducting and selecting segregating populations [6, 7]. The diallel also allows the breeder to know the *per se* behavior of the parents, called general combining ability (GCA), besides their hybrid combinations, called specific combining ability (SCA). GCA is attributed to genes with additive effects, whereas SCA is related to non-additive gene effects, which characterizes the difference of the hybrid combinations concerning the average behavior of the parent [8]. Hence, this approach enables to select segregating populations with high SCA for the traits of interest, and that includes at least one of the parents having high GCA [9].

Soybean crop has a restriction regarding the use of plants in F_1_ generation for diallel analysis, due to the low availability of seeds. This problem can be overcome by using the F_2_ generation [10–12]. However, for each self-fertilization generation advanced from F_1_, the contribution of the dominance deviation in the population mean is halved. This condition may contribute to the SCA effect to be insignificant in subsequent generations and cause loss of information on gene complementation among the parents used [13]. In this sense, the use of partial diallel may be more appropriate, since in this diallel the magnitude of GCA, besides quantifying the frequency of favorable alleles, indicates the genetic diversity between the parent from one group and those from the opposite group [14]. The analysis in advanced generations of a partial diallel is justified because of the possibility of bias in GCA estimates due to the predominance of dominance deviations when F_1_ generation is used [15].

The use of diallel analysis in the F_2_ generation has been applied to the breeding of several crops, such as wheat [10, 16, 13], soybean [11, 17, 18] and common bean [19, 20]. The study of the combining ability of genotypes, besides providing valuable information to the decision making about the choice of parents, allows the understanding of the genetic action involved in the trait inheritance, contributing to greater efficiency of soybean breeding programs.

As already mentioned, obtaining high yielding genotypes is the main goal of soybean breeding. However, increased photosynthetic efficiency has become the target of several breeding programs [21–23]. The positive correlation between the photosynthetic rate and the yield in soybean suggests that the improvement of photosynthetic efficiency may be a promising target for new yield gains [22, 24, 25]. Zhu et al. [26] estimated that at least a 50% improvement in photosynthetic efficiency will also be critical to meet the doubled global yield of grain crops over this century. Morgan et al. [27] and Dermody et al. [28] provide evidence that increasing photosynthesis in a crop under standard field production conditions does result in increased yield. In these experiments, when photosynthesis was increased by artificial elevation of CO_2_, there was an increase in yield of 15%.

In addition to photosynthetic capacity, other traits such as stomatal conductance, internal CO_2_ concentration, and transpiration are essential for understanding plant physiological metabolism and identifying genotypes with greater photosynthetic and water-use efficiencies. Wong et al. [29] reported that the photosynthetic capacity is correlated with gs, suggesting that genotypes with higher gs have a higher photosynthetic rate. In turn, genotypes with lower transpiration rates have greater water-use efficiency. In this sense, genotypes with higher gs and photosynthetic rate and lower transpiration can be promising in the formation of populations in breeding programs aiming at both yield increase and resistance to abiotic stress.

Although they are crucial in traditional breeding aimed at photosynthetic efficiency, there are no studies on combining ability of soybean crop for physiological traits to date. Therefore, we performed a diallel analysis in soybean lines and F_2_ populations aiming to i) estimate the combining ability of genotypes based on F_2_ generation, and ii) identify superior segregating parents and populations for physiological traits.

## 2. Material and methods

### 2.1. Obtaining progenies in F_1_ generation

The hybrids were obtained in a greenhouse from the Soybean Program of the Department of Plant Science at Federal University of Viçosa (20º45’14"S; 42º52’53"W, 649 m of altitude), from October 2016 to January 2017. For assembly of the crossing blocks, we selected contrasting parents for flower color, in which the males carried alleles for purple flower (dominant) and females carried alleles for white flower (recessive). The divergence regarding the relative maturity group (RMG) was also taken as the selection criteria of parents (Table 1).

**Table 1:** Characteristics of the 11 soybean genotypes used as parents in each group: Flower color and relative maturity group (RMG).

Genotype	Flower color	RMG
**Group I (male parents)**		
**BMX Prisma IPRO**	Purple	7.5
**M6952 IPRO**	Purple	7.2
**BMX Bônus IPRO**	Purple	7.9
**BMX Flecha IPRO**	Purple	6.6
**M6410 IPRO**	Purple	6.4
**NS 6909 IPRO**	Purple	6.9
**M7739 IPRO**	Purple	7.7
**Group II (female parents)**		
**BMX Ponta IPRO**	White	6.1
**DM 6563 RSF IPRO**	White	6.3
**SYN 13671 IPRO**	White	7.1
**TMG 7062 IPRO**	White	6.2

Based on the mentioned characteristics, 11 transgenic parents (carrier of Intacta RR2 PRO™ technology) were selected and divided into two groups: male (group I) and female (group II).

### 2.2. Obtaining progenies in F_2_ generation

The 28 F_1_ hybrids were carried out in a greenhouse from the Soybean Program of the Department of Plant Science at Federal University of Viçosa (20º45’14"S; 42º52’53"W, 649 m of altitude) from February 2017 to June 2017. Hybrid seeds were sown in a 3 L plastic pot, and one plant per pot was maintained after the identification of hybrid plants characterized by purple flower. The control of weeds, pests and diseases was carried out according to technical recommendations for the crop.

### 2.3. Conducting progenies in F_2_ generation

**Fig 1:**
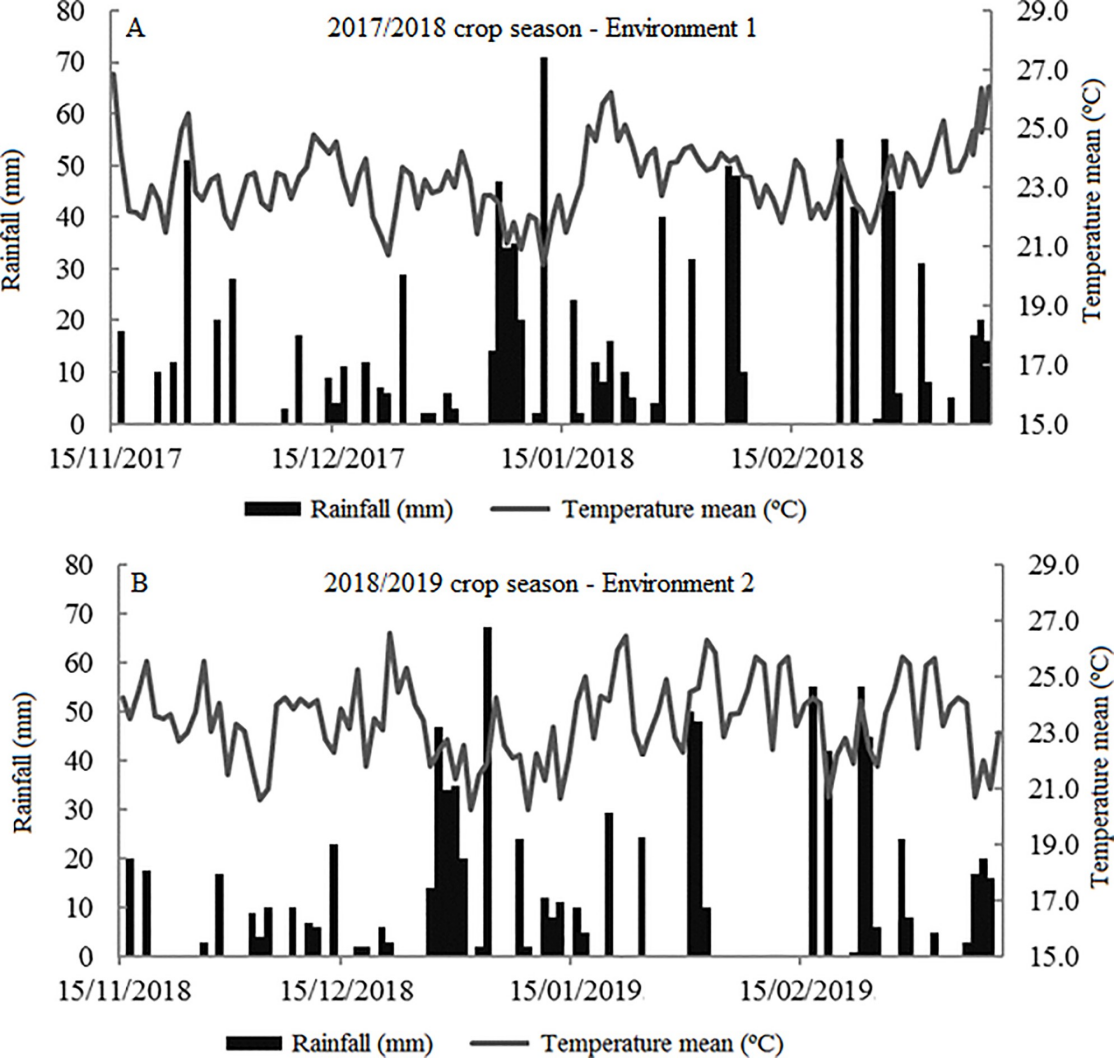
Climatic conditions observed during the 2017/2018 and 2018/2019 crop seasons.

Two experiments (2017/2018 and 2018/2019 crop seasons) evaluating the 28 F_2_ populations were carried out in the experimental field at Federal University of Mato Grosso do Sul, Campus Chapadão do Sul (18°46'26"S, 52°37'28"W and an average altitude of 810 m). The design used was an extended complete block with two replicates due to the low availability of seeds to conduct the experiments in two environments (crop seasons). The experimental unit consisted of three lines for each F_2_ population. The control of weeds, pests and diseases was carried out according to technical recommendations for the crop. Phytosanitary management followed the recommendations for soybean cultivation. The climatic conditions observed over each experiment are shown in Fig 1.

### 2.4. Traits evaluated in F_2_ generation

At 60 days after emergence (DAE), physiological traits were analyzed using a portable photosynthesis analyzer (*Infrared Gas Analyzer*—IRGA) model Li-6400XT (LiCor Inc., Lincoln, Nebraska, USA). Photosynthetically active photon flux of 1044 μmol m^-2^ s^-1^ and environment CO_2_ concentrations (372 ± 10 mol m^-2^ s^-1^) were used, according to similar studies evaluating gas exchange in soybean crop [30, 31]. The physiological traits measured were: net photosynthesis (A, mmol CO_2_ m^-2^ s^-1^), transpiration (E, mmol H_2_O m^-2^ s^-1^), stomatal conductance (gs, mmol m^-2^ s^-1^), and internal CO_2_ concentration (Ci, mmol m^-2^ s^-1^).

In both crop seasons, the measurements were carried out between 9:00 h and 10:00 h a.m. in five plants randomly sampled from each experimental unit. Measurements were taken on cloudless days with temperatures between 26.0 and 26.5ºC, and relative humidity between 50 and 80%. We used the third leaf fully developed from the apex of the plant, which is considered diagnostic for soybean nutritional analysis [32], and this is where occurs most of the metabolic processes responsible for the energy acquisition by plants.

### 2.5. Statistical analysis

Initially, a joint analysis of variance was performed according to the statistical model described below:

$$Y_{ijk}=\mu +B/E_{jk}+G_{i}+E_{j}+GxE_{ij}+e_{ijk}$$ (1)

Wherein: Y_ijk_ is the observation in the k-th block, evaluated in the i-th genotype and j-th environment (crop season); μ is the overall mean of the experiments; B/E_jk_ is the effect of k block within the j environment; G_i_ is the effect of the i-th genotype considered as fixed; E_j_ is the effect of the j-th environment considered as random; GxE_ij_ is the random effect of the interaction between i genotype and j environment; e_ijk_ is the random error associated with observation Y_ijk_.

After verifying that the interaction between genotypes x environments was not significant, the partial diallel analysis was performed according to the Griffing model [33], adapted to partial diallel by Geraldi and Miranda Filho [34]. The treatment effect, considered as fixed, was decomposed into general combining ability (GCA) and specific combining ability (SCA) according to the statistical model described in Eq 2.

$$Y_{ij}=\mu +\frac{1}{2}(d_{1}+d_{2})+g_{i}+g_{j}+S_{ij}+\bar{\varepsilon }$$ (2)

Wherein: *Y_ij_* is mean involving the i-th parent from Group I and the j-th parent from Group II; *μ* is the overall mean of the diallel; *d*_1_ and *d*_2_ are contrasts involving means of the Groups I and II and the overall mean; *g_i_* is the effect of the general combining ability of the i-th parent from Group I; *g_j_* is the effect of the general combining ability of the j-th parent from Group II; *S_ij_* is the effect of specific combining ability; and $\bar{\varepsilon }$ is the mean random error.

For purposes of a proper interpretation of the results, the significance of GCA and SCA estimates were assessed by t-test. Thus, we considered only the estimates that differed from zero, that is, which were significant by t-test at 5% probability level. The sum of squares of treatments was unfolded into sum of GCA squares of the groups I and II and SCA. The magnitude of additive and non-additive effects was inferred by the ratio between sums of GCA mean squares (Groups I + II) and SCA mean squares, since the mean square has no orthogonal decomposition [35]. All statistical analysis was performed with the Genes software [36], following the procedures recommended by Cruz et al. [9].

## 3. Results

### 3.1. Diallel analysis of variance

There was a significant effect (P <0.05) of genotypes for all physiological traits (Table 2). These results indicate the existence of genetic variability among genotypes for the evaluated traits. The effects of genotypes were unfolded into GCA effects of Groups I and II and SCA. The GCA mean squares of Groups I and II were significant for all traits. Similar to the GCA effects, there was a significant effect of SCA for all the traits. It is important to highlight that the GxE interaction was not significant for all evaluated traits. It is possible to note that the means for all traits presented by the populations, although close to the parents, were slightly higher than those obtained by the parents. The coefficients of variation (CV) were less than 4%, revealing high experimental accuracy and data reliability.

**Table 2:** Mean squares from diallel analysis for net photosynthesis (A), stomatal conductance (gs), internal CO_2_ concentration (Ci) and transpiration (E), evaluated in F_2_ populations and their parents grown in 2017/2018 and 2018/2019 crop seasons.

Sources of variation	DF	A	gs	Ci	E
**Blocks/Environment**	2	0.04	0.06	43.85	0.22
**Genotypes (G)**	34	23.22*	0.19*	1010.42*	0.82*
**GCA–Group I**	3	46.38*	0.32*	720.16*	0.51*
**GCA–Group II**	6	74.89*	0.24*	1218.28*	2.04*
**SCA**	28	25.86*	0.24*	1149.90*	1.11*
**Environment (E)**	1	123.11*	5.57*	5934.16*	10.29*
**GxE**	34	0.44^ns^	0.02^ns^	25.34^ns^	0.06^ns^
**GCAxE–Group I**	3	1.25^ns^	0.02^ns^	15.16^ns^	0.03^ns^
**GCAxE–Group II**	6	0.98^ns^	0.01^ns^	10.90^ns^	0.02^ns^
**SCAxE**	28	0.44^ns^	0.02^ns^	17.33^ns^	0.03^ns^
**Error**	20	0.39	0.01	16.33	0.04
**Means–Parents**		54.88	1.16	416.34	6.78
**Means–Populations**		55.53	1.39	437.78	7.29
**CV (%)**		1.14	3.85	0.94	2.75

^ns^ and *: not significant and significant at 5% probability by F test, respectively

DF: degrees of freedom; CV: coefficient of variation.

As mentioned, the sum of squares between GCA (Groups I and II) and SCA was adopted as a criterion for assessing the magnitude of genic effects. Based on this criterion, the GCA mean squares were higher than the SCA mean squares for all evaluated traits. Hence, additive effects are predominant in controlling these traits.

### 3.2. Combining ability of parents and F_2_ populations

**Fig 2:**
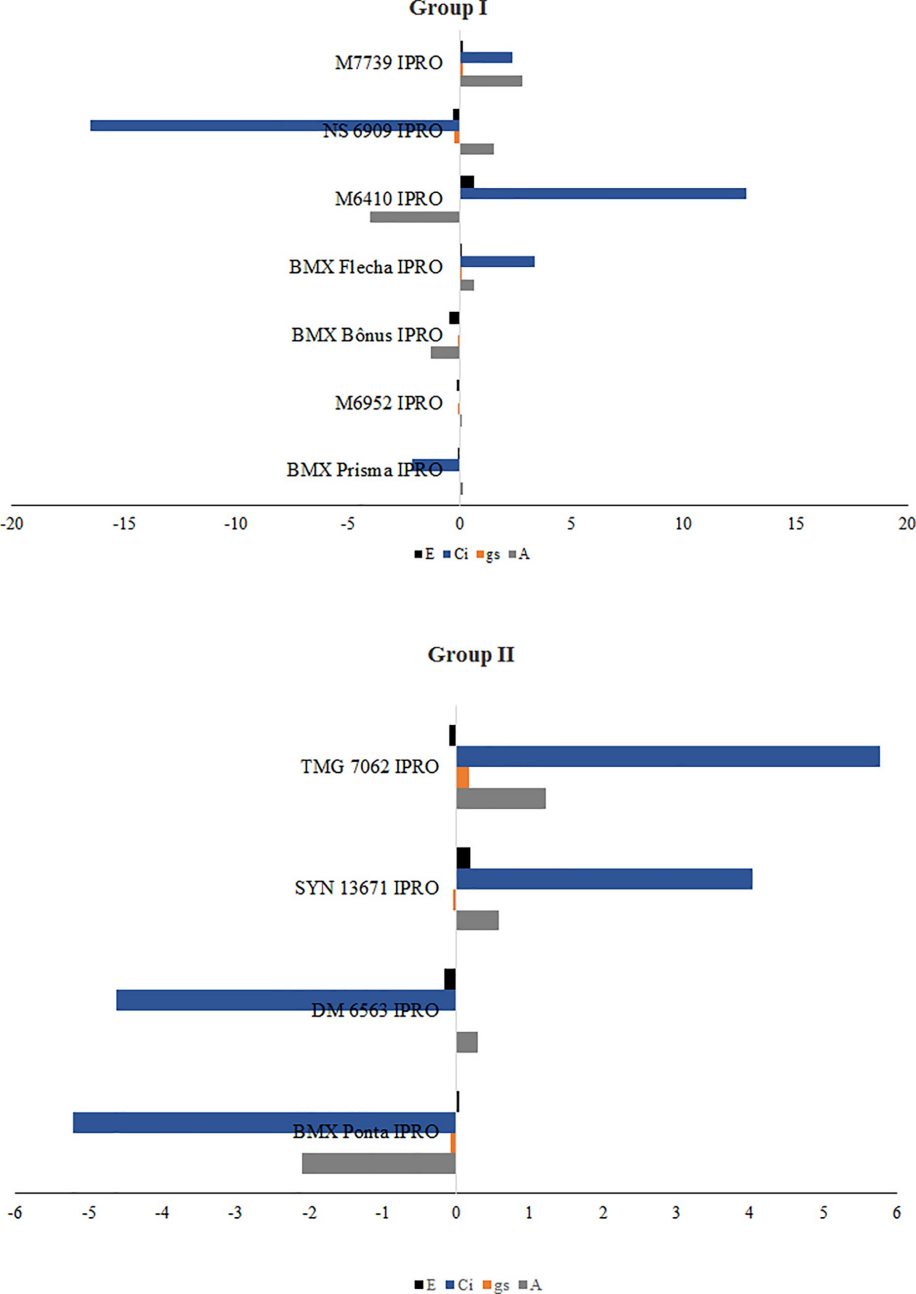
General combining ability estimates of the parents from Groups I and II for net photosynthesis (A, mmol CO_2_ m^-2^ s^-1^), transpiration (E, mmol H_2_O m^-2^ s^-1^), stomatal conductance (gs, mmol m^-2^ s^-1^), and internal CO_2_ concentration (Ci, mmol m^-2^ s^-1^).

**Fig 3:**
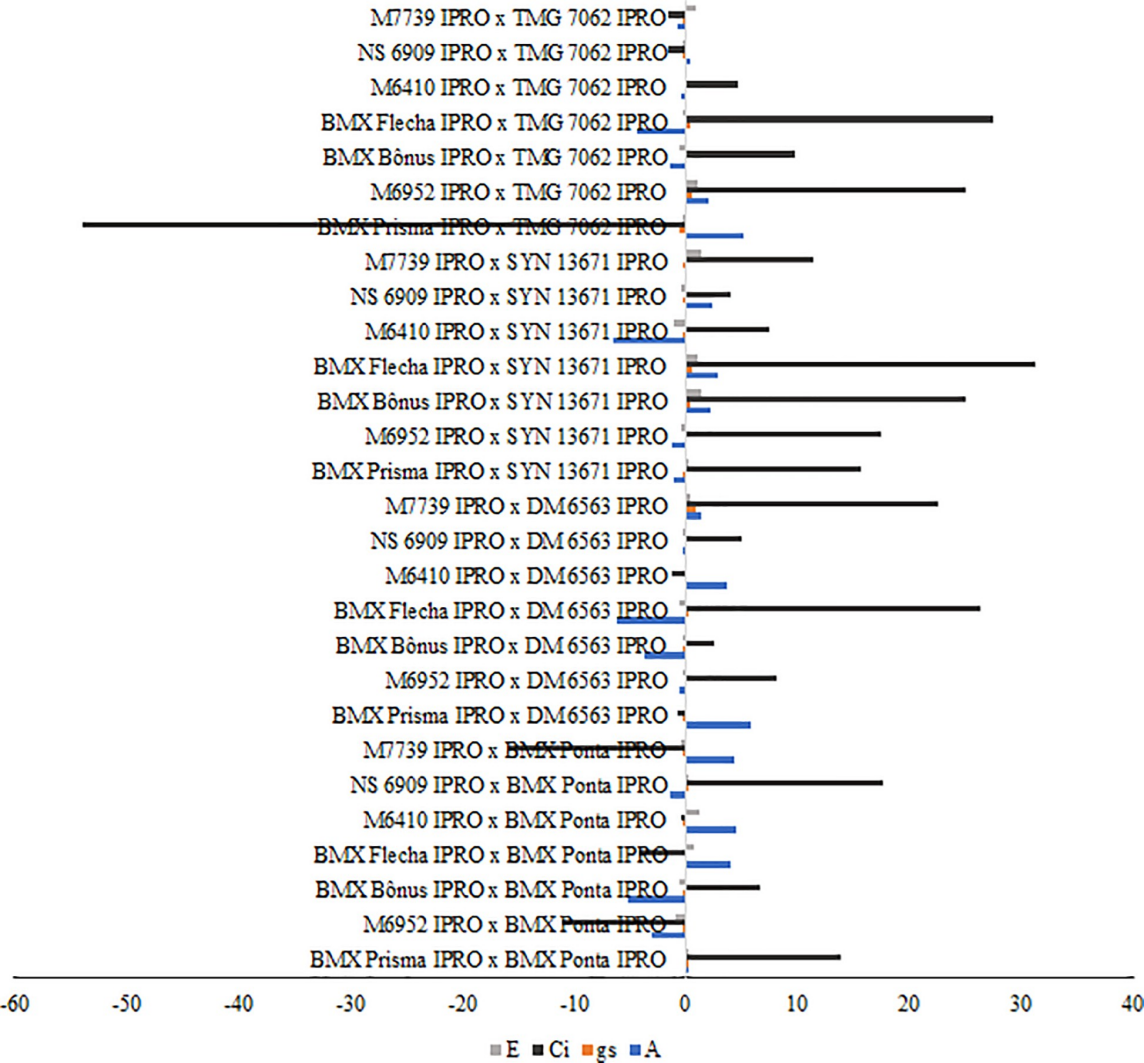
Specific combining ability estimates of F_2_ populations for net photosynthesis (A, mmol CO_2_ m^-2^ s^-1^), transpiration (E, mmol H_2_O m^-2^ s^-1^), stomatal conductance (gs, mmol m^-2^ s^-1^), and internal CO_2_ concentration (Ci, mmol m^-2^ s^-1^).

The GCA estimates of the parents from Groups I and II are shown in Fig 2. The GCA and SCA estimate values and their significance (if the values differ from zero) by t-test are available in S1 and S2 Tables, respectively. Regarding the net photosynthesis (A), the highest GCA estimates for Group I were obtained by the parents NS 6909 IPRO and M7739 IPRO, whereas for Group II the highest estimate was observed for the parent TMG 7062 IPRO. Among the highest SCA estimates for A, the populations M7739 IPRO x BMX Ponta IPRO, NS 6909 IPRO x SYN 13671 IPRO, BMX Prisma IPRO x TMG 7062 IPRO and M6952 IPRO x TMG 7062 IPRO can be highlighted by presenting at least one parent with high GCA for the trait (Fig 3).

Significant GCA estimates for gs were observed for the parents BMX Flecha IPRO and M7739 IPRO (Group I), and TMG 7062 IPRO (Group II). The populations BMX Flecha IPRO x DM 6563 IPRO, M7739 IPRO x DM 6563 IPRO, BMX Flecha IPRO x SYN 13671 IPRO, M7739 IPRO x SYN 13671 IPRO, M6952 IPRO x TMG 7062 IPRO and BMX Flecha IPRO x TMG 7062 IPRO stood out because they had the highest SCA estimates and at least one parent with higher GCA estimates.

For the internal CO_2_ concentration (Ci), the parents BMX Flecha IPRO, M6410 IPRO and M7739 IPRO (Group I) and SYN 13671 IPRO and TMG 7062 IPRO (Group II) presented significant GCA estimates. The populations BMX Flecha IPRO x DM 6563 IPRO, M7739 IPRO x DM 6563 IPRO, BMX Prisma IPRO x SYN 13671 IPRO, M6952 IPRO x SYN 13671 IPRO, BMX Bônus IPRO x SYN 13671 IPRO, BMX Flecha IPRO x SYN 13671 IPRO, M7739 IPRO x SYN 13671 IPRO, M6952 IPRO x TMG 7062 IPRO, and BMX Flecha IPRO x TMG 7062 IPRO had the highest SCA estimates associated with high GCA estimates of at least one of their parents. Finally, the parents with negative GCA estimates for transpiration (E) were M6952 IPRO, BMX Bônus IPRO and NS 6909 IPRO (Group I) and DM 6563 IPRO (Group II). The highest SCA estimates for E were obtained by the populations M6952 IPRO x BMX Ponta IPRO and M6410 IPRO x SYN 13671 IPRO, and only the population M6952 IPRO x BMX Ponta IPRO presented one of their parents with lower SCA estimates.

Based on the results obtained for A, gs and Ci, the genotype TMG 7062 IPRO stood out with desirable GCA and SCA estimates for these traits. Other genotypes that can be highlighted are SYN 13671 IPRO and BMX Flecha IPRO for internal CO_2_ concentration, and M7739 IPRO for net photosynthesis. It is worth mentioning that although the parent M6410 IPRO presented the highest GCA estimate for Ci of the Group I, none of the cross-breeding involving this parent had satisfactory SCA estimates. Hence, we do not recommend the use of M6410 IPRO as parent in crossing blocks aiming at obtaining offspring with higher Ci.

## 4. Discussion

### 4.1. Understanding of gene effects controlling physiological traits

The coefficients of variation (CV) less than 4% show high experimental accuracy and are lower than the values reported in the literature [30, 31, 37, 38]. After conducting the experiments in two crop seasons, it was observed that the genotype x environment interaction was not significant for all traits evaluated. These results were expected, since the phytosanitary management performed in the crop seasons were the same. Moreover, the climatic conditions that occurred in both crop seasons were very similar (Fig 1). Total rainfall in the first crop season was 675 mm, while in the second crop season it was 643 mm. The average temperature in the first crop season was 26.6ºC, while in the second crop season it was 26.4ºC.

In cases where GCA is significant, it can be inferred that at least one of the parents differs from the others regarding the concentration of favorable alleles [9, 13]. Hence, the significance of GCA effects is indicative of the existence of parents who contribute to a greater number of favorable alleles for these traits to be transmitted to offspring [39].

Significant SCA effects reveal that there are deviations in the behavior of the hybrids compared to what was expected based on the parent's GCA [8]. The GCA is attributed to genes with additive effects, whereas SCA is related to non-additive gene effects [9]. Therefore, the presence of significant GCA and SCA effects show the importance of both additive and non-additive genetic components controlling the studied traits.

Based on the diallel analysis, it is possible to evaluate the relative importance of additive gene effects (expressed by GCA effects), as well as the effects due to dominance (associated with SCA). This information is useful in establishing the best breeding strategy [5]. The GCA mean squares higher than the SCA mean squares reveal the predominance of additive effects on the control of all traits, although non-additive effects may also be involved [6]. There are still no studies in the literature evaluating the genetic effects involved in the control of traits A, gs, Ci and E in soybean. Our study is the first to elucidate the predominance of additive effects controlling these traits, revealing that the selection of parents based on these traits is promising.

When additive effects are pronounced, gains of greater magnitude will be predicted [5]. Additive effects of genes are cumulative over generations and are the main sources of genetic variation exploited by most breeding programs [40], since it is responsible for setting the traits of interest.

In this sense, the selection based on the physiological traits evaluated here, which are useful in soybean breeding programs aiming at photosynthetic efficiency, can be carried out at initial generations due to the predominance of additive effects in F_2._ This generates time savings in the evaluation and conduction of populations, contributing to greater efficiency of breeding programs.

### 4.2. Combining ability estimates to identify parents and promising populations for breeding of physiological traits

The photosynthetic rate is positively correlated with soybean yield [22, 23, 37, 38]. Todeschini et al. [25], when evaluating the genetic progress of a historical cultivar set in South Brazil released between 1965 and 2011, they verified that the photosynthetic rate, transpiration rate, and chlorophyll a and b content improved significantly over the years and were positively associated with seed yield. Therefore, photosynthetic efficiency is related not only to net photosynthesis but also to the mechanisms of stomatal conductance, internal CO_2_ concentration and transpiration. For this reason, it is important to understand the combining ability for these traits in order to select parents who have alleles favorable to be transmitted to their offspring.

Stomatal conductance (gs) is a measure of the relationship between the passage of carbon dioxide (CO_2_) entering and the water vapor flowing through the leaf stomata [41]. Under non-limiting conditions for water availability and environment temperatures below thermal stress levels, the maximal stomatal conductance of a genotype will maximize the photosynthetic rates [29, 42]. This is because the first response to a water deficit is the change in gs, thereby limiting photosynthesis [23, 43]. Roche [41] also report that the CO_2_ uptake maintenance, which is required by high photosynthetic rates at times of the day with high irradiation, contributes to a higher final yield.

The importance of gs in the initial responses to water stress has been reported in the literature [41–44]. As gs is related to leaf turgor and this, in turn, depends on the balance between water loss through transpiration and water supply to the leaf from the soil [45], it can be inferred that gs is directly related with transpiration. In this sense, genotypes with greater CO_2_ assimilation and lower transpiration rates are desirable, since they will show greater water-use efficiency.

In this sense, identifying genotypes with great combining ability for A, Ci, gs and E is crucial to guide crossbreeding step. Based on the results obtained for combining ability, we can verify a superiority of the genotype TMG 7062 IPRO for A, gs and Ci. This fact can also be confirmed when observing the performance of its progeny resulting from the cross-breeding M6952 IPRO x TMG 7062 IPRO, which presented high SCA estimates for these traits. Due to its great combining ability for the mentioned traits, the genotype TMG 7062 IPRO shows to be promising for use as parent in breeding programs for obtaining populations with greater photosynthetic and water-use efficiencies.

Traditional breeding prevails in soybean programs in Brazil, having the use of diallel crosses and phenotypic information as the main approaches used in the choice of parents and formation of base-population. Furthermore, soybean breeding programs are still very lacking in studies on physiological traits, and thus most of them do not take into account the measurement of these parameters for evaluating parents and progenies. Our findings revealed that there are differences between genotypes regarding the concentration of favorable alleles for Ci, gs, A and E. The diallel analysis also allowed to identify the predominant genetic effects controlling the evaluated traits.

Todeschini et al. [25], in a study assessing the genetic progress of several agronomic, phenological and physiological traits in South Brazil, they reported that breeding strategies which maximize the photosynthetic rate, transpiration rate and chlorophyll content may increase the genetic progress for soybean yield in the future. Therefore, the evaluation of A, Ci, gs and E, which are easy to measure and low cost, can be valuable for soybean breeding programs, since it can contribute simultaneously to greater photosynthetic efficiency and yield gains [26]. Given the findings already reported in the literature and the results found here, we recommend using the traits net photosynthesis, stomatal conductance, internal CO_2_ concentration and transpiration rate in indirect selection on soybean grain yield, due to the positive association between photosynthetic capacity and yield, as well as the additive nature of the traits, which ensures greater gains from selection.

However, we also understand that further studies on population choice based on combining ability for physiological traits, especially other traits not evaluated here, are needed for a better understanding of genetic effects involved in the control of traits related to photosynthetic and water-use efficiencies. Such studies may contribute to clarify which physiological traits are critical for identifying promising lines, as well as provide information about the genetic effect of the traits, which will guide the crossing and progeny selection steps.

## 5. Conclusions

By analyzing combining ability of soybean genotypes for net photosynthesis, stomatal conductance, internal CO_2_ concentration, and transpiration, we identified a predominance of additive effects controlling the traits. This finding is important for setting and guiding strategies to be adopted in soybean breeding programs using these traits in the choice of genotypes for base-population. Since additive effects are predominant on the traits evaluated here, higher-magnitude genetic gains with selection on these traits will be predicted, since they are responsible for setting the traits of interest.

The diallel analysis also allowed to identify superior parents and segregating populations for physiological traits. The genotype TMG 7062 IPRO presented high GCA estimates for A, gs and Ci, and given these results, it can be used as parent for crosses to improve photosynthetic efficiency. Lastly, populations coming from the cross-breeding between SYN 13671 IPRO and TMG 7062 IPRO, with BMX Flecha IPRO and BMX Bonus IPRO present higher potential for obtaining superior lines for photosynthetic and water-use efficiencies.

## Supporting information

S1 Table Values used for diallel analysis of photosynthesis (A), stomatal conductance (gs), internal CO_2_ concentration (Ci) and transpiration (E) obtained in F_2_ populations of soybean grown in 2017/2018 and 2018/2019 crop seasons.
(DOCX)

S2 Table Values used for diallel analysis of photosynthesis (A), stomatal conductance (gs), internal CO_2_ concentration (Ci) and transpiration (E) obtained in parents of soybean grown in 2017/2018 and 2018/2019 crop seasons.
(DOCX)
