# Supplementary material for: Understanding the combining ability for physiological traits in soybean
Source: PLoS One. 2019 Dec 17;14(12):e0226523. doi: 10.1371/journal.pone.0226523 (PMC6917344; doi:10.1371/journal.pone.0226523)
Supplement: S1 Table — Values used for diallel analysis of photosynthesis (A), stomatal conductance (gs), internal CO2 concentration (Ci) and transpiration (E) obtained in F2 populations of soybean grown in 2017/2018 and 2018/2019 crop seasons. (DOCX) [file pone.0226523.s001.docx]

**S1 Table.** Values used for diallel analysis of photosynthesis (A), stomatal conductance (gs), internal CO_2_ concentration (Ci) and transpiration (E) obtained in F_2_ populations of soybean grown in 2017/2018 and 2018/2019 crop seasons.

| **Male Parents** | **Female Parents** | **A** | **gs** | **Ci** | **E** |
| --- | --- | --- | --- | --- | --- |
| BMX Prisma IPRO | BMX Ponta IPRO | 55.92 | 1.48 | 445.45 | 7.53 |
| M6952 IPRO | BMX Ponta IPRO | 53.23 | 1.02 | 422.78 | 6.27 |
| BMX Bônus IPRO | BMX Ponta IPRO | 49.64 | 1.15 | 440.41 | 6.26 |
| BMX Flecha IPRO | BMX Ponta IPRO | 60.70 | 1.40 | 432.85 | 8.24 |
| M6410 IPRO | BMX Ponta IPRO | 56.58 | 1.28 | 446.06 | 9.12 |
| NS 6909 IPRO | BMX Ponta IPRO | 56.16 | 1.29 | 434.93 | 7.32 |
| M7739 IPRO | BMX Ponta IPRO | 63.27 | 1.17 | 420.17 | 7.02 |
| BMX Prisma IPRO | DM 6563 RSF IPRO | 62.62 | 1.49 | 432.57 | 7.02 |
| M6952 IPRO | DM 6563 RSF IPRO | 56.19 | 1.59 | 443.65 | 6.76 |
| BMX Bônus IPRO | DM 6563 RSF IPRO | 51.73 | 1.35 | 438.09 | 6.39 |
| BMX Flecha IPRO | DM 6563 RSF IPRO | 51.15 | 1.83 | 465.05 | 6.59 |
| M6410 IPRO | DM 6563 RSF IPRO | 56.45 | 1.53 | 446.99 | 7.79 |
| NS 6909 IPRO | DM 6563 RSF IPRO | 58.08 | 1.35 | 424.01 | 6.66 |
| M7739 IPRO | DM 6563 RSF IPRO | 60.92 | 2.50 | 460.31 | 7.59 |
| BMX Prisma IPRO | SYN 13671 IPRO | 52.54 | 1.18 | 438.10 | 7.45 |
| M6952 IPRO | SYN 13671 IPRO | 52.31 | 1.33 | 441.93 | 6.64 |
| BMX Bônus IPRO | SYN 13671 IPRO | 54.26 | 1.62 | 449.56 | 8.09 |
| BMX Flecha IPRO | SYN 13671 IPRO | 56.89 | 1.86 | 459.10 | 8.30 |
| M6410 IPRO | SYN 13671 IPRO | 43.00 | 0.99 | 444.81 | 6.75 |
| NS 6909 IPRO | SYN 13671 IPRO | 57.29 | 1.00 | 411.94 | 6.57 |
| M7739 IPRO | SYN 13671 IPRO | 56.23 | 1.17 | 438.17 | 8.74 |
| BMX Prisma IPRO | TMG 7062 IPRO | 61.12 | 0.74 | 369.10 | 6.65 |
| M6952 IPRO | TMG 7062 IPRO | 57.94 | 1.73 | 450.11 | 7.85 |
| BMX Bônus IPRO | TMG 7062 IPRO | 53.07 | 1.25 | 434.84 | 5.90 |
| BMX Flecha IPRO | TMG 7062 IPRO | 52.14 | 1.69 | 455.95 | 7.07 |
| M6410 IPRO | TMG 7062 IPRO | 51.32 | 1.29 | 442.46 | 7.68 |
| NS 6909 IPRO | TMG 7062 IPRO | 57.62 | 1.00 | 407.06 | 6.55 |
| M7739 IPRO | TMG 7062 IPRO | 57.88 | 1.18 | 425.79 | 8.01 |
