# Supplementary material for: Understanding the combining ability for physiological traits in soybean
Source: PLoS One. 2019 Dec 17;14(12):e0226523. doi: 10.1371/journal.pone.0226523 (PMC6917344; doi:10.1371/journal.pone.0226523)
Supplement: S2 Table — Values used for diallel analysis of photosynthesis (A), stomatal conductance (gs), internal CO2 concentration (Ci) and transpiration (E) obtained in parents of soybean grown in 2017/2018 and 2018/2019 crop seasons. (DOCX) [file pone.0226523.s002.docx]

**S2 Table.** Values used for diallel analysis of photosynthesis (A), stomatal conductance (gs), internal CO_2_ concentration (Ci) and transpiration (E) obtained in parents of soybean grown in 2017/2018 and 2018/2019 crop seasons.

| **Parent** | **A** | **gs** | **Ci** | **E** |
| --- | --- | --- | --- | --- |
| BMX Prisma IPRO | 49.99 | 1.72 | 453.06 | 6.63 |
| M6952 IPRO | 56.02 | 1.17 | 425.13 | 6.76 |
| BMX Bônus IPRO | 55.78 | 1.09 | 422.73 | 5.9 |
| BMX Flecha IPRO | 57.51 | 1.13 | 410.99 | 6.49 |
| M6410 IPRO | 45.81 | 1.66 | 465.16 | 7.97 |
| NS 6909 IPRO | 56.85 | 0.91 | 399.18 | 6.44 |
| M7739 IPRO | 57.42 | 1.73 | 441.21 | 6.08 |
| BMX Ponta IPRO | 56.11 | 1.08 | 419.31 | 7.61 |
| DM 6563 RSF IPRO | 58.75 | 0.83 | 394.89 | 7.52 |
| SYN 13671 IPRO | 52.82 | 0.65 | 347.97 | 6.46 |
| TMG 7062 IPRO | 56.74 | 0.83 | 400.37 | 6.76 |
